# Geospatial inequalities and determinants of caesarean section delivery in sub-Saharan Africa: a multi-country analysis

**DOI:** 10.1080/16549716.2026.2686564

**Published:** 2026-06-18

**Authors:** Belayneh Jejaw Abate, Eliyas Addisu Taye, Endalew Minwuye Andargie, Halima Ayalew Kebede, Helen Brhan Alemaw, Sofiya Ayalew Kebede, Alo Edin, Martha Solomon Tadesse, Angefa Ayele, Amare Teshome Tefera

**Affiliations:** a University of Gondar Comprehensive Specialized Hospital, Gondar, Ethiopia; b Department of Health Informatics, College of Medicine and Health Sciences, University of Gondar Comprehensive Specialized Hospital, Gondar, Ethiopia; c Department of Public Health, School of Public Health, Asrat Woldeyes Health Science Campus, Debre Berhan University, Debre Berhan, Ethiopia; d Department of Public Health, College of Health Sciences, Woldia University, Woldia, Ethiopia; e Department of Epidemiology and Biostatistics, School of Public Health, College of Medicine and Health Sciences, Wollo University, Dessie, Ethiopia; f Department of Epidemiology, School of Public Health, Institute of Health, Bule Hora University, Bule Hora, Ethiopia; g Department of Pediatrics and Child Health, School of Medicine, College of Medicine and Health Science, University of Gondar, Gondar, Ethiopia; h Department of Dentistry, School of Medicine, College of Medicine and Health, Science, University of Gondar, Gondar, Ethiopia

**Keywords:** Caesarean section delivery, geospatial analysis, lifesaving intervention, multi-scale geographic weighted regression, sub-saharan Africa

## Abstract

**Background:**

Cesarean section is a lifesaving obstetric intervention when medically indicated; however, its utilization remains unequal across sub-Saharan Africa (SSA). Although the World Health Organization recommends cesarean section rates of 10–15%, access remains insufficient in many low-resource settings and excessive in others. Understanding geographic patterns and drivers is essential for maternal health planning.

**Objective(s):**

To examine the spatial variation and determinants of cesarean section delivery across SSA.

**Methods:**

We conducted a cross-sectional analysis using Demographic and Health Survey data (2015–2024) from 201,481 weighted samples across 28 SSA countries. Spatial autocorrelation and hotspot patterns were assessed using Global Moran’s I and Getis-Ord Gi* statistics. Spatial regression models, including ordinary least squares, spatial lag, spatial error, geographically weighted regression, and multiscale geographically weighted regression, were fitted. Model performance was compared using corrected Akaike Information Criterion and adjusted R^2^.

**Results:**

Cesarean section delivery showed significant spatial clustering (Moran’s I = 0.18, z = 43.3, *p* < 0.01). Hotspot areas were identified in Uganda, Rwanda, Burundi, Kenya, Tanzania, Malawi, South Africa, Lesotho, Gabon, Ghana, and Senegal, while cold spots were observed in Ethiopia, Madagascar, Angola, Nigeria, Guinea, Cote d’Ivoire, Sierra Leone, Liberia, and Mauritania. Previous cesarean delivery, maternal age ≥35 years, pregnancy spacing behavior, and health insurance coverage were significant spatial predictors.

**Conclusion:**

Cesarean section utilization in SSA exhibits substantial geographic inequality driven by context-specific determinants. Spatially targeted maternal health policies, improved referral systems, and equitable financing mechanisms are needed to optimize access to medically indicated cesarean delivery while minimizing unnecessary procedures.

## Paper Context

- **Main findings:** Caesarean section utilization in SSA demonstrated substantial geographic inequalities, with significant spatial clustering and regional variation influenced by previous caesarean sections, maternal age, insurance coverage, and reproductive health behaviors.
- **Added knowledge:** This study provides multicounty geospatial evidence using multiscale geographically weighted regression to identify location-specific predictors and geographic inequalities in caesarean section utilization across SSA.
- **Global health impact for policy and action:** Geographically targeted maternal health strategies are needed to improve equitable access to medically necessary caesarean section services while preventing unnecessary procedures across underserved regions of SSA.

## Background

Caesarean section delivery is a critical intervention in modern obstetrics. As a major surgical procedure to deliver a baby, it serves as a vital tool for reducing maternal and neonatal mortalities by addressing life-threatening complications during pregnancy and labor [1].

Globally, around 21% of all births are currently delivered by caesarean section, increasing from about 12% in 2000, and are projected to reach 29% by 2030 [2]. This trend is largely driven by increases in non-medically indicated procedures in many middle- and high-income countries [3–5]. However, this global average masks profound disparities, with caesarean section (C-section) rates in some high-income regions exceeding 40%, while in many parts of sub-Saharan Africa, they remain below 5% [6].

Evidence shows that a C-section rate exceeding 20% does not correlate with improved perinatal or neonatal outcomes at the population level [5,7,8]. In contrast, numerous low- and middle-income countries still have C-section rates below 10%, indicating limited access to medically necessary procedures [7,8]. Taking this into account, significant guidance has been established through organizations like the World Health Organization, which recommends an optimal C-section rate of 10–15% for population-level health [7,9]. Initiatives like the Robson Classification have been introduced to audit and rationalize C-section use, aiming to reduce unnecessary procedures while ensuring access for those in need [2,10]. Despite this progress, achieving equitable access without overuse remains a major challenge in sub-Saharan Africa (SSA).

Previous studies from SSA countries have identified various predictors associated with higher C-section delivery, including gestational age of less than 37 weeks, maternal underweight body mass index, previous uterine surgery, obstetrical complications, birth weight of 3500 g or more, lack of antenatal care, difficulty accessing health facilities, no history of vaginal delivery, advanced maternal age, parity, women education status, wealth index, and cultural practices affecting maternal and neonatal care [11–15]. However, these studies often rely on relative measures of association such as odds ratios (ORs) and relative risks (RRs), which do not account for geographic variability, spatial patterns, or interactions influenced by location. This limitation hinders the detection of spatial dependence among neighboring areas and identification of geographic clusters of under-service or overuse of C-section delivery [16,17]. As a result, health planners lack detailed, location-specific evidence critical for efficient resource allocation and targeted public health interventions. Thus, traditional non-spatial regression and aggregated national statistics are insufficient for nuanced public health planning and resource distribution in the context of C-section delivery. Furthermore, previous spatial analyses were typically limited to single countries without accounting for variation scales across predictors [18–20]. Geospatial techniques like multi-scale geographically weighted regression (MGWR), by contrast, analyze data at various spatial scales, enabling a more flexible understanding of how influencing factors change geographically, an insight that traditional methods may miss [21,22]. In light of these gaps, our study aims to examine the spatial distribution and determinants of C-section delivery across SSA using geospatial analysis. The findings will provide an actionable evidence base for targeting interventions in the most underserved communities, ultimately advancing the dual goals of improving access to life-saving surgery and preventing unnecessary procedures.

## Methods

### Study design, period, and setting

This study utilized publicly available cross-sectional datasets from the Demographic and Health Surveys (DHS) program, conducted between 2015 and 2024 across 28 SSA countries. These surveys offer valuable data on a wide range of health and demographic indicators in low- and middle-income countries. Countries were grouped into four regions based on the most recent DHS data: Eastern SSA (Burundi, Ethiopia, Malawi, Kenya, Tanzania, Mozambique, Rwanda, Uganda, Zambia, Zimbabwe), western SSA (Benin, Burkina Faso, Ivory Coast, Liberia, Mali, Gambia, Ghana, Guinea, Mauritania, Nigeria, Senegal, Sierra Leone), Central SSA (Cameroon, Angola, Gabon), and Southern SSA (Lesotho, Madagascar, South Africa) (Supplementary File 1). The datasets are publicly accessible through the DHS Program website (www.dhsprogram.com).

### Sampling procedure and sample size determination

The DHS dataset utilized a two-stage stratified sampling method. In the first stage, clusters were selected using a probability proportional to the size of each area, based on the variable v001 from individual country datasets. Each country’s clusters were sequentially numbered to prevent overlaps; for instance, Ethiopia’s 645 clusters were labeled from 1 to 645, and the next country began numbering at 646. This systematic arrangement was consistently applied across all countries, resulting in a total of 17,416 enumeration areas in the first stage. In the second stage, a fixed number of households within each cluster were systematically chosen equally. Women between 15 and 49 years old, including permanent residents and visitors who stayed overnight in the selected households prior to the day of data collection, were eligible to participate in the interviews. This study utilized a weighted sample comprising 201,481 women of reproductive age from the DHS Individual Records datasets across the 28 countries who had a history of childbirth during the study period (Supplementary File 2).

### Study variables

#### Dependent variable

The outcome variable for this study was C-section delivery. This was derived from a DHS survey question with binary responses, which were coded as 0 for ‘No’ and 1 for ‘Yes’ for analysis.

#### Independent variables

This study examined several independent variables, including age of respondent, place of residence, media exposure, educational attainment, age of respondent, wealth index, preceding birth interval, birth order, history of terminated pregnancy, anything used to delay pregnancy, number of children in the household, birth weight, source of drinking water, last birth by C-section, age at the first birth, distance to health facility, preterm birth status, antenatal care (ANC) attendance, decision-making power, and insurance coverage. Details on the measurement of each predictor were displayed in supplementary material (Supplementary File 3).

### Statistical analysis

Prior to analysis, data quality was assessed, and the handling of missing values and sample weights was addressed. Missing data were detected in several variables, which ranged from 0.09% (age at first birth) to 4.24% (husband’s occupation). The pattern of missingness was arbitrary (Supplementary File 4, Table 1) and assumed to be missing at random (MAR) (Supplementary File 4, Table 2). In accordance with DHS guidelines, missing values were handled using the recommended procedures detailed in Supplementary File 4, Table 3. To account for unequal sample selection probabilities, sample weights were applied using the svyset Stata command before conducting any statistical analyses, as recommended by the DHS guideline [23].

### Spatial analysis

The spatial analysis started with the acquisition of two key data sources: a continental shape file of Africa from African Boundaries (https://hub.arcgis.com/datasets/geoduck:africa-boundaries/explore) and the geospatial data for enumeration areas from the MEASURE DHS program (www.dhsprogram.com). We cleaned each country’s dataset individually, assigned new cluster numbers within each dataset, and then merged them. These merged datasets were combined with the coordinate files to create a unified dataset. Finally, the unified dataset was then projected using the Albers Equal Area Conic projection in ArcGIS 10.1.

In this study, geographic inequality refers to measurable spatial differences in C-section utilization across geographic locations in SSA. It was assessed using a sequential spatial analytical framework that included global spatial autocorrelation analysis, hotspot and cold spot identification, cluster and outlier detection, spatial interpolation, and spatial regression analyses. These approaches were used to determine whether C-section utilization was geographically clustered and to identify factors associated with the observed spatial variation.

To identify the spatial pattern of C-section delivery in SSA, we employed a suite of spatial analyses. Global Moran’s I was used to assess whether C-section delivery was clustered, dispersed, or randomly distributed across SSA [24,25]. While Moran’s I identifies overall clustering patterns, it does not indicate which regions have high or low C-section utilization.

To identify regions with significantly high or low C-section utilization, Getis-Ord Gi* statistics were applied, using Z-scores and corresponding *p*-values to determine statistical significance. A high positive z-score with a statistically significant *p*-value indicates a geographic clustering of high C-section utilization (hot spot), whereas a low negative z-score with a statistically significant *p*-value indicates a geographic clustering of low C-section utilization (cold spot) [26]. However, hotspot analysis does not identify spatial outliers.

To address this limitation, a cluster and outlier analysis was performed. This method identifies significant clusters and spatial outliers, complementing the hotspot analysis. Nonetheless, it does not provide information on relative risk, log likelihood, radius of coverage, or prediction in unsampled areas. Spatial scan statistics were employed to identify statistically significant primary and secondary clusters and estimate their relative risks and geographic extent, whereas ordinary kriging interpolation was used to predict C-section utilization in unsampled areas [27,28]. Detailed descriptions of the spatial analytical procedures are presented in Supplementary File 5 A to E.

While the preceding analyses identify spatial patterns of C-section utilization, they do not explain how the relationships between C-section delivery and its predictors vary geographically. To investigate factors associated with the observed spatial variation, we adopted a sequential regression modeling approach.

We first fitted an Ordinary Least Squares (OLS) regression model as a baseline global regression model to identify potential predictors and assess model assumptions. Exploratory regression was conducted to evaluate multicollinearity, normality, stationarity, and spatial independence. Since OLS assumes uniform relationships across space, Spatial Lag Models (SLM) and Spatial Error Models (SEM) were introduced to address spatial dependencies. The Lagrange Multiplier (LM) test guided model selection: significant LM-lag and robust LM-lag tests support SLM, while significant LM-error and robust LM-error tests support SEM (Supplementary File 6, Figure 1). Both tests were significant (*p* < 0.001), leading to the use of SLM and SEM to capture spatial influence from neighboring regions and spatial autocorrelation in residuals [29,30]. The analysis was performed using GeoDa software.

**Figure 1.**
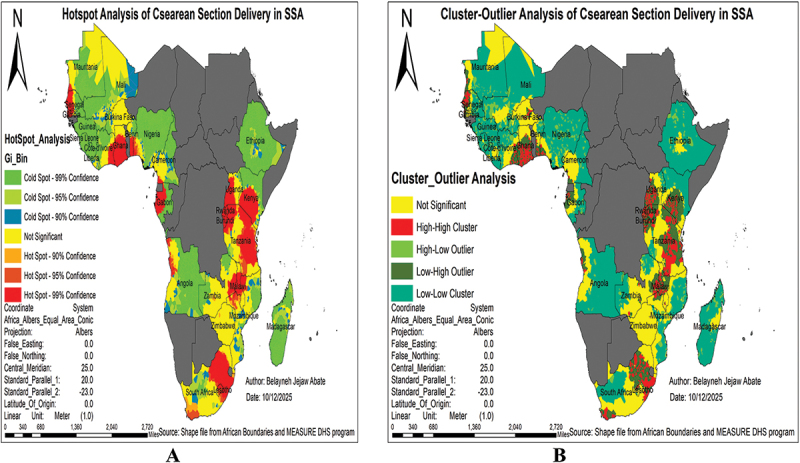
Hotspot (1A) and cluster-outlier (1B) analysis of caesarean section delivery in SSA: DHS 2015–2024.

Red color shades in Figures 1 A and B indicate countries with high caesarean section delivery, and green color shades indicate countries with low caesarean section delivery. Strength of presence is indicated by depth of color. SSA denotes sub-Saharan Africa, DHS denotes demographic and health survey data.

The significant Koenker-Breusch-Pagan test revealed non-stationarity in the relationship between C-section delivery and predictors, prompting the use of Geographically Weighted Regression (GWR) to account for spatial variability. However, GWR assumes a single spatial scale for all variables, a limitation addressed by Multiscale Geographically Weighted Regression (MGWR). MGWR advances GWR by determining variable-specific bandwidths through a golden section search, allowing each predictor to function at its optimal spatial scale. Additionally, MGWR employs an iterative back-fitting procedure during calibration, capturing varying spatial scales of relationships and improving the model’s ability to account for different spatial processes [31,32]. Details on the spatial regression models are presented in Supplementary File 6.

The study evaluated global (OLS) and local (GWR, MGWR) models by comparing their corrected Akaike information criteria (AICc) and adjusted R^2^ values. The optimal model was selected based on the lowest AICc and highest adjusted R^2^ values. Significant C-section delivery predictors identified in the final model were geographically mapped to highlight country-level variations.

### Ethical considerations

This study utilized publicly available de-identified datasets obtained from the Demographic and Health Surveys (DHS) Program. Permission to access and use the datasets was obtained from the DHS Program. Ethical approval for data collection was secured by the respective national health research ethics committees and institutional review boards in each participating country prior to survey implementation. Informed consent was obtained from all participants during the original surveys. The present study was conducted in accordance with the ethical principles outlined in the Declaration of Helsinki. Furthermore, this study followed the Strengthening the Reporting of Observational Studies in Epidemiology (STROBE) guidelines for cross-sectional studies (STROBE Checklist) [33].

## Results

### Characteristics of study participants

Our analysis incorporated nationally representative data from 201,481 reproductive-aged women with a history of childbirth across 28 SSA countries. Nearly half (48.24%) of the women were between 25 and 34 years old. Most participants resided in rural areas (64.79%) and were identified as poor (42.95%). While most women had some form of education, a substantial proportion (34.37%) had no formal education. Additionally, 65.39% of the women reported exposure to media. Furthermore, a large majority (93.02%) indicated that their last delivery was not by caesarean section. Detailed descriptive statistics of the predictors are provided in Supplementary File 7.

### Global spatial autocorrelation (Moran’s I) analysis

The study found significant spatial clustering (Moran’s I 0.14, z-score 143.7, *p*-value < 0.01), revealing a 99% confidence level in the clustering pattern of C-section delivery across the regions of SSA (Supplementary File 8). Getis-Ord General G analysis confirmed this clustering with a z-score of 116.8 and a *p*-value less than 0.001, showing that high values are more concentrated than expected by chance (Supplementary File 9). This finding indicates that C-section utilization is spatially clustered rather than randomly distributed across SSA.

### Hot spot analysis of caesarean section delivery in SSA

The Getis-Ord Gi* analysis identified statistically significant spatial clusters of high and low C-section utilization across SSA. Areas with high positive z-scores and statistically significant *p*-values were classified as hot spots (clusters of high C-section utilization), while areas with low negative z-scores and statistically significant *p*-values were classified as cold spots (clusters of low C-section utilization).

Hot spots of C-section utilization were observed in Uganda, Rwanda, Burundi, Kenya, Tanzania, Malawi, South Africa, Lesotho, Gabon, Ghana, and Senegal. Cold spots were identified in Ethiopia, Madagascar, Angola, Nigeria, Guinea, Côte d’Ivoire, Sierra Leone, Liberia, and Mauritania (Figure 1A).

### Cluster-outlier analysis of caesarean section delivery in SSA

Significant high–high clusters were detected in Kenya, Uganda, Rwanda, Burundi, Tanzania, Malawi, Lesotho, and Ghana. In contrast, low–low significant clusters were reported in Ethiopia, Madagascar, Angola, Nigeria, Guinea, Sierra Leone, and Mauritania. The study also identified low–high outliers in Senegal, Gabon, and Burkina Faso, as well as high–low outliers in Cote d’Ivoire and Liberia (Figure 1B).

### Spatial prediction of caesarean section delivery in SSA

**Figure 2.**
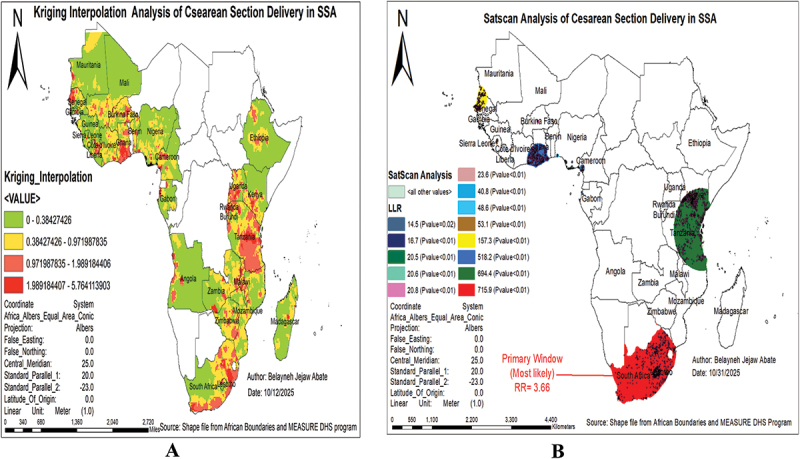
Kriging interpolation (2A) and satscan (2B) analysis of caesarean section delivery in SSA: DHS 2015–2024.

The ordinary kriging spatial interpolation method indicated that areas predicted to have higher rates of C-section delivery were represented in red, while those with lower rates are shown in green. Consequently, Uganda, Rwanda, Burundi, Kenya, Tanzania, Malawi, Lesotho, Zimbabwe, Ghana, Burkina Faso, and Senegal were identified as areas with predicted higher C-section utilization. Whereas, areas with predicted lower C-section utilization for C-section delivery were found in Ethiopia, Madagascar, Angola, Nigeria, Mali, Liberia, Sierra Leone, Guinea, Gabon, and Mauritania (Figure 2A).

Red color shades in Figures 2 A and B indicate countries with high caesarean section delivery, and green color shades indicate countries with low caesarean section delivery. Strength of presence is indicated by depth of color. SSA denotes sub-Saharan Africa, DHS denotes demographic and health survey data, C.I. denotes confidence interval, RR denotes relative risk, and LLR denotes log likelihood ratio.

### SatScan analysis of caesarean section delivery in SSA

The Kulldorff spatial scan statistical analysis identified a total of 3,867 significant clusters of C-section deliveries, comprising 1,146 primary clusters and 2,721 secondary clusters. The most prominent cluster (primary window) is located in South Africa and Lesotho, with geographical coordinates of (33.861285°S, 18.728133°E) and a radius of 1,654.70 km. Women residing in these primary clusters were found to be 3.66 times more likely to experience C-section delivery compared to those living outside the area (RR = 3.66, LLR = 715.9, *p*-value < 0.01) (Figure 2B). Further details on the significant secondary clusters can be found in Supplementary File 10.

### Spatial predictors of caesarean section delivery in SSA

C-section delivery was spatially clustered (Moran’s I 0.14, z-score 143.6, *p*-value < 0.01) across regions of SSA, indicating a violation of the ordinary least squares (OLS) assumption of spatial independence. This led us to utilize a spatial dependence model. The significant Lagrange multiplier tests (*p* < 0.001) for both lag and error effects prompted two additional global spatial models (SLM and SEM) to better account for the underlying spatial dependence; all predictors were checked on the assumption of spatial dependence (Supplementary File 11). However, the significant Koenker-Basset statistics enabled us to perform local regression models (GWR and MGWR). Among all models, MGWR accounted for up to 65.1% of the variability related to C-section deliveries in SSA. This is evidenced by the decrease in the Akaike Information Criterion corrected (AICc) from −6672.2 for the OLS model to −33179 for the MGWR model, indicating that MGWR is the most appropriate model (Supplementary File 12).

In the MGWR model, all predictors were statistically significant, exhibiting varying spatial effects with optimal bandwidths ranging from 68 to 17,280, allowing adaptation to each variable’s unique spatial features. In contrast, the GWR model uses a fixed bandwidth of 2.87 for all predictors, failing to capture the different spatial scales at which each predictor operates (Supplementary File 13).

**Figure 3.**
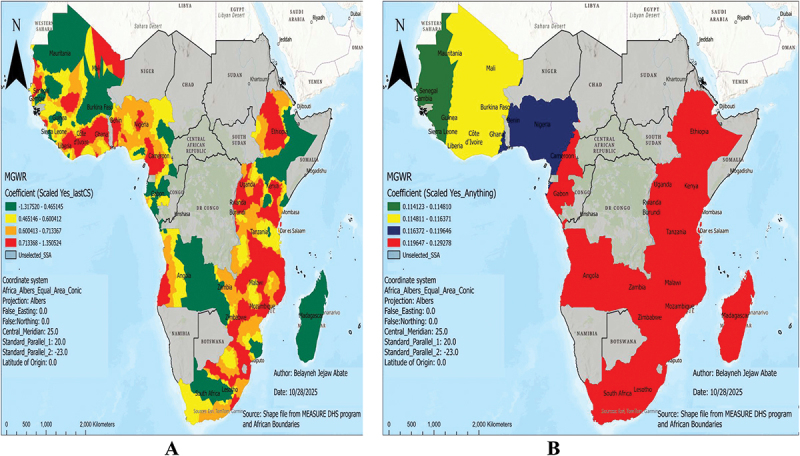
MGWR result of last birth by caesarean section coefficients (A) and anything used to delay or avoid pregnancy (B) for determining caesarean section delivery in SSA, DHS 2015–2024.

**Figure 4.**
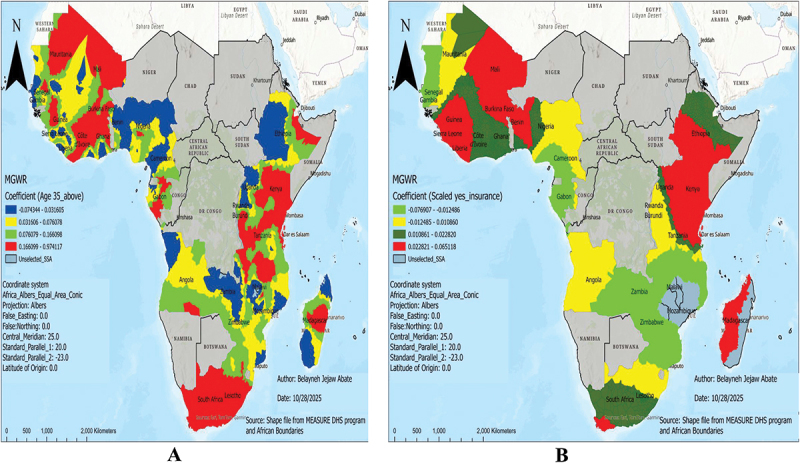
MGWR result of age of respondent 35 and above (A) and insurance coverage (B) for determining caesarean section delivery in SSA, DHS 2015–2024.

The MGWR output identified several significant spatial predictors of C-section deliveries in SSA. These include a previous C-section for the last birth, efforts to delay or avoid pregnancy, being 35 years or older, and residing in areas with insurance coverage. These findings are illustrated in Figures 3 and 4.

Coefficient estimates are obtained from MGWR regression by utilizing ArcGiS Pro version 3 software. Predictors included are last birth by caesarean section (Yes_lastCS and anything used to delay or avoid pregnancy (Yes_Anything). Red color shades in indicate countries with high caesarean section delivery associated with selected predictors. Strength of presence is indicated by depth of color. DHS denotes demographic and health survey data and MGWR represents multi-scale geographic weighted regression.

Coefficient estimates are obtained from MGWR regression by utilizing ArcGiS Pro version 3 software. Predictors included are age of respondent 35 and above (Age_35_above) and insurance coverage (Yes_Insurance). Red color shades in indicate countries with high caesarean section delivery associated with selected predictors. Strength of presence is indicated by depth of color. DHS denotes demographic and health survey data and MGWR represents multi-scale geographic weighted regression.

Last births by C-section delivery were statistically geographically associated with C-section delivery in areas of SSA. A 10% increase in women delivering their last child by C-section resulted in a 7.1% to 13.5% rise in C-section delivery in areas of Ethiopia, Kenya, Rwanda, Uganda, Burundi, Nigeria, Côte d’Ivoire, Malawi, Mozambique, Ghana, and Liberia (Figure 3A).

A 10% increase in attempts to delay or avoid pregnancy is associated with a 1.2% to 1.3% increase in C-section delivery in significant areas of Ethiopia, Kenya, Rwanda, Uganda, Burundi, Tanzania, Zambia, Malawi, Mozambique, Zimbabwe, Madagascar, South Africa, Lesotho, Angola, and Gabon (Figure 3B).

In significant areas, a 10% increase in respondents who were 35 years and above resulted in a 1.6% to 9.7% increase in C-section delivery in areas of Kenya, Madagascar, Lesotho, Mauritania, Ghana, Burkina Faso, Mali, and South Africa (Figure 4A).

Insurance coverage was a significant spatial predictor of C-section delivery in SSA. In significant areas, a 10% increase in women utilizing insurance coverage is associated with a 0.2% to 0.5% rise in C-section delivery in areas of Ethiopia, Kenya, Madagascar, Uganda, Mali, Burkina Faso, Benin, Guinea, Sierra Leone, and Liberia (Figure 4B).

## Discussion

Spatial analysis is crucial for identifying geographic disparities in health service utilization and guiding context-specific interventions to ensure equitable maternal care across SSA [34,35]. In this study, we assessed the spatial distribution and determinants of C-section delivery using nationally representative DHS data from 2015 to 2024 across 28 SSA countries. Our findings revealed substantial spatial heterogeneity in C-section delivery, with significant clustering patterns and varying determinants across regions.

Our findings revealed significant spatial autocorrelation (Moran’s I = 0.14, *p* < 0.01), indicating that C-section delivery rates are geographically clustered across SSA. Hot spots were found in Uganda, Rwanda, Burundi, Kenya, Tanzania, Malawi, Lesotho, and South Africa. Conversely, cold spots were identified in Ethiopia, Madagascar, Angola, Nigeria, Guinea, Sierra Leone, and Mauritania. These findings suggest that access to and utilization of C-section services varies considerably across geographic locations. Such variation may reflect differences in the availability of comprehensive emergency obstetric care, distribution of skilled health professionals, accessibility of health facilities, socioeconomic conditions, and health system capacity across countries and regions [3,5,36]. Regions with well-developed health infrastructures [37,38], higher urbanization, and greater concentrations of health professionals tended to exhibit higher C-section rates, whereas infrastructural barriers, geographic inaccessibility, and financial constraints plausibly contribute to underutilization in other areas [39,40]. These findings align with the World Health Organization’s (WHO) assertion that both underuse and overuse of C-section coexist globally, but underuse remains a predominant challenge in SSA [9,41,42]. Our findings provide new evidence that C-section delivery is unevenly distributed across SSA and highlight the need for national and regional health policies to prioritize cold spot regions with persistently low C-section rates.

Spatial prediction through ordinary kriging further highlighted areas with elevated probabilities of C-section delivery, primarily concentrated in East and Southern Africa. These findings emphasize the value of spatial modeling in identifying high-priority regions for targeted intervention [43,44]. Health planners can utilize such predictive maps to allocate surgical resources effectively, enhance maternal referral systems, and strengthen regional obstetric services.

Our study revealed that in a previous C-section, efforts to delay or avoid pregnancy, advanced maternal age, and insurance coverage were significant spatial predictors of c-section delivery

Regions with the highest coefficients for a previous C-section, such as Ethiopia, Kenya, Rwanda, Uganda, Nigeria, and Ghana, showed a strong positive association with current C-section rates. The possible justification might be the standard clinical practice of recommending a repeat CS after a prior one, driven by limited access to and support for Vaginal Birth after Caesarean (VBAC) in many SSA settings [5,45,46]. Additionally, provider preferences and health system protocols may favor scheduled repeat caesareans for clinical convenience or risk management. The observed pattern may also reflect constraints on clinical capacity, risk management strategies, or adherence to standardized obstetric protocols. Prior obstetric complications, including obstructed labor or fetal distress, further elevate the likelihood of a repeat C-section. The finding that a previous C-section is a strong predictor of subsequent C-section delivery aligns with existing evidence, reflecting clinical guidelines and health system dynamics [5,45–47]. This underscores the need for health systems to strengthen vaginal birth after caesarean capacity, review repeat-CS protocols, and implement strategies that balance maternal safety with avoidance of unnecessary surgery.

Regions with higher coefficients of attempts to delay or avoid pregnancy, including Ethiopia, Kenya, Rwanda, Uganda, Burundi, Tanzania, Zambia, Malawi, Mozambique, Zimbabwe, Madagascar, South Africa, Lesotho, Angola, and Gabon, exhibit elevated C-section rates. This pattern may reflect higher levels of health-seeking behavior, contraceptive use, and access to facility-based obstetric care among women who actively manage their fertility. Additionally, women who delay or limit childbearing often face higher obstetric risks, further increasing the likelihood of caesarean deliveries. Moreover, stronger health systems and urban populations, where enhanced clinical capacity and provider preferences further contribute to the higher use of caesarean sections [5,10,43,48]. The findings highlight the need for comprehensive reproductive health strategies that integrate family planning services with evidence-based obstetric care.

In regions such as Kenya, Madagascar, Lesotho, Mauritania, Ghana, Burkina Faso, Mali, and South Africa, a higher proportion of women aged 35 years and above was associated with increased C-section rates. This trend likely reflects the elevated obstetric risks associated with advanced maternal age, including conditions like preeclampsia, gestational diabetes, malpresentation, and prolonged labor, which often require surgical delivery [5,10]. Older mothers are also more likely to utilize facility-based maternity care and to receive C-section delivery as a precautionary measure due to perceived or actual risks [48,49]. Clinicians should adopt more cautious delivery approaches for older mothers, favoring caesarean sections to minimize potential complications [46]. The regional discrepancies might also highlight variations in health system capacity, clinical practices, and socioeconomic profiles that influence the likelihood of surgical delivery [5,6,46]. The findings highlight the need for targeted maternal health strategies that strengthen risk assessment, ensure evidence-based clinical decisions, and promote safe delivery practices for older mothers.

Insurance coverage has emerged as a significant spatial predictor of C-section delivery across sub-Saharan Africa. In areas such as Ethiopia, Kenya, Madagascar, Uganda, Mali, Burkina Faso, Benin, Guinea, Sierra Leone, and Liberia, higher utilization of insurance coverage was associated with increased C-section rates. This relationship likely reflects improved financial access to facility-based maternity care and skilled birth attendance among insured women, which enhances the likelihood of surgical delivery when medically indicated [50,51]. Insurance facilitates hospital delivery and access to surgical services, leading to higher use of C-sections when clinically indicated. However, it may also encourage provider-driven practices in systems where reimbursement incentives exist. Moreover, insured women are more likely to deliver in better-equipped facilities where C-section services are available, and providers may adopt lower thresholds for surgical intervention to manage perceived risk [51,52]. This pattern emphasizes that expanding financial protection mechanisms is essential for improving access to maternal health care.

A key strength of this study is the use of large, nationally representative datasets, ensuring robust and generalizable findings. The application of a suite of complementary spatial statistics and an advanced multiscale regression model provides a nuanced and sophisticated understanding of the spatial dynamics of C-section delivery. However, these findings should be interpreted considering certain limitations. First, the use of cross-sectional data prevents the establishment of causal relationships. Second, the analysis relies on secondary data, which may lack detailed clinical information on the specific indications for each C-section (e.g. fetal distress, obstructed labor, and maternal request), making it difficult to distinguish between medically necessary and potentially unnecessary procedures. Third, the geographical locations of clusters in the DHS are adjusted for confidentiality, with enumeration areas shifted by up to 2 km in urban settings and 5 km in rural areas; while this may slightly affect the precision of cluster-level analysis, the use of large-scale spatial trends helps mitigate this concern.

## Conclusion

C-section utilization in SSA exhibits significant geographic inequalities, characterized by marked spatial clustering and regional variation. The observed patterns are influenced by context-specific determinants, including previous C-section delivery, attempts to delay or avoid pregnancy, advanced maternal age, and insurance coverage. Addressing these disparities requires targeted interventions in low-utilization areas, strengthening obstetric services, improving referral systems, and expanding financial access to ensure equitable, medically indicated C-section delivery across the region.

## Supplementary Material

Supplementary file CS_rev.docx

STROBEchecklist.doc

## Data sharing statement

The analysis utilized data from the Demographic and Health Surveys (DHS). Detailed information about the datasets and their contents is available at https://dhsprogram.com/data/available-datasets.cfm

## References

[1] Biccard BM, Madiba TE, Kluyts H-L, et al. Perioperative patient outcomes in the African surgical outcomes study: a 7-day prospective observational cohort study. Lancet. 2018;391:1589–13. doi: 10.1016/S0140-6736(18)30001-1 29306587

[2] Betran AP, Ye J, Moller A-B, et al. Trends and projections of caesarean section rates: global and regional estimates. BMJ Glob Health. 2021;6:e005671. doi: 10.1136/bmjgh-2021-005671 PMC8208001 34130991

[3] Betran AP, Torloni MR, Zhang J, et al. What is the optimal rate of caesarean section at population level? A systematic review of ecologic studies. Reprod Health. 2015;12:57. doi: 10.1186/s12978-015-0043-6 26093498 PMC4496821

[4] Ye J, Betrán AP, Guerrero Vela M, et al. Searching for the optimal rate of medically necessary cesarean delivery. Birth. 2014;41:237–244. doi: 10.1111/birt.12104 24720614

[5] Betrán AP, Ye J, Moller A-B, et al. The increasing trend in caesarean section rates: global, regional and national estimates: 1990–2014. PLoS One. 2016;11:e0148343. doi: 10.1371/journal.pone.0148343 26849801 PMC4743929

[6] Wells JC, Wibaek R, Poullas M. Global epidemiology of use of and disparities in caesarean sections. Lancet. 2019;394:24–25. doi: 10.1016/S0140-6736(19)30715-9 31282355

[7] Betrán AP, Torloni MR, Zhang J-J, et al. Who statement on caesarean section rates. BJOG. 2015;123:667. 26681211 10.1111/1471-0528.13526 PMC5034743

[8] Molina G, Weiser TG, Lipsitz SR, et al. Relationship between cesarean delivery rate and maternal and neonatal mortality. JAMA. 2015;314:2263–2270. doi: 10.1001/jama.2015.15553 26624825

[9] World Health Organization. Who statement on caesarean section rates. Geneva: World Health Organization; 2015.

[10] Vogel JP, Betrán AP, Vindevoghel N, et al. Use of the Robson classification to assess caesarean section trends in 21 countries: a secondary analysis of two WHO multicountry surveys. Lancet Glob Health. 2015;3:e260–e70. doi: 10.1016/S2214-109X(15)70094-X 25866355

[11] Irwinda R, Hiksas R, Lokeswara AW, et al. Maternal and fetal characteristics to predict c-section delivery: a scoring system for pregnant women. Womens Health (Lond). 2021;17:17455065211061969. PubMed PMID: 34818932; PubMed Central PMCID: PMCPMC8785277. doi: 10.1177/17455065211061969 PMC8785277 34818932

[12] Agimas MC. Prevalence of emergency caesarean delivery and its predictors among women who give birth in Ethiopia using further analysis of EDHS 2016 data: a mixed effect model. PLoS One. 2024;19:e0300528. Epub 20240516. PubMed PMID: 38753832; PubMed Central PMCID: PMCPMC11098499. doi: 10.1371/journal.pone.0300528 PMC11098499 38753832

[13] Ushie BA, Udoh EE, Ajayi AI. Examining inequalities in access to delivery by caesarean section in Nigeria. PLoS One. 2019;14:e0221778. Epub 20190829. PubMed PMID: 31465505; PubMed Central PMCID: PMCPMC6715280. doi: 10.1371/journal.pone.0221778 PMC6715280 31465505

[14] Islam MA, Sathi NJ, Hossain MT, et al. Caesarean delivery and its association with educational attainment, wealth index, and place of residence in sub-Saharan Africa: a meta-analysis. Sci Rep. 2022;12:5554. Epub 20220401. PubMed PMID: 35365718; PubMed Central PMCID: PMCPMC8975863. doi: 10.1038/s41598-022-09567-1 PMC8975863 35365718

[15] Mezemir R, Olayemi O, Dessie Y. Trend and associated factors of cesarean section rate in Ethiopia: evidence from 2000–2019 Ethiopia demographic and health survey data. PLoS One. 2023;18:e0282951. Epub 20230316. PubMed PMID: 36928080; PubMed Central PMCID: PMCPMC10019649. doi: 10.1371/journal.pone.0282951 PMC10019649 36928080

[16] Song Y, Su Y, Wang Z. Variable selection of spatial logistic autoregressive model with linear constraints. Entropy (Basel). 2022;24:1600. Epub 20221115. PubMed PMID: 36421516; PubMed Central PMCID: PMCPMC9689031. doi: 10.3390/e24111660 PMC9689031 36421516

[17] Getu K, Gangadhara Bhat H. Application of geospatial techniques and binary logistic regression model for analyzing driving factors of urban growth in Bahir Dar city, Ethiopia. Heliyon. 2024;10:e25137. doi: 10.1016/j.heliyon.2024.e25137 38322870 PMC10844060

[18] Ayele M, Lake ES, Tilahun BD, et al. Geographically weighted regression analysis of cesarean delivery using the Ethiopian mini demographic and health survey 2019. Sci Rep. 2025;15:5338. doi: 10.1038/s41598-025-87962-0 39948376 PMC11825925

[19] Sy I, Sandie AB, Sylla EM, et al. Spatial and socioeconomic inequalities in cesarean section deliveries in urban settings in Dakar, Senegal. J Urban Health. 2024;101:81–91. doi: 10.1007/s11524-024-00835-1 38507023 PMC11602882

[20] Mohammadi A, Pishgar E, Salari Z, et al. Geospatial analysis of cesarean section in Iran (2016–2020): exploring clustered patterns and measuring spatial interactions of available health services. BMC Preg Childbirth. 2022;22:582. Epub 20220721. PubMed PMID: 35864462; PubMed Central PMCID: PMCPMC9302231. doi: 10.1186/s12884-022-04856-z PMC9302231 35864462

[21] Fotheringham AS, Yang W, Kang W. Multiscale geographically weighted regression (MGWR). Ann Am Assoc Geogr. 2017;107:1247–1265. doi: 10.1080/24694452.2017.1352480

[22] Li Z, Fotheringham AS. Computational improvements to multi-scale geographically weighted regression. Int J Geogr Inf Sci. 2020;34:1378–1397. doi: 10.1080/13658816.2020.1720692

[23] DHS Program. Guide to DHS statistics; 2023 [cited 2024]. p. 8. Available from: https://dhsprogram.com/pubs/pdf/DHSG1/Guide_to_DHS_Statistics_DHS-8.pdf

[24] Chen Y. New approaches for calculating Moran’s index of spatial autocorrelation. PLoS One. 2013;8:e68336. doi: 10.1371/journal.pone.0068336 23874592 PMC3709922

[25] Delele TG, Persson LÅ, Schellenberg J, et al. Geographic equity in essential newborn care practices in Ethiopia: a cross-sectional study. BMC Pediatr. 2025;25:281. doi: 10.1186/s12887-025-05645-1 40205458 PMC11983989

[26] Tsai P-J, Lin M-L, Chu C-M, et al. Spatial autocorrelation analysis of health care hotspots in Taiwan in 2006. BMC Public Health. 2009;9:464. doi: 10.1186/1471-2458-9-464 20003460 PMC2799414

[27] Bhunia GS, Shit PK, Maiti R. Comparison of GIS-based interpolation methods for spatial distribution of soil organic carbon (SOC). J Saudi Soc Agric Sci. 2018;17:114–126. doi: 10.1016/j.jssas.2016.02.001

[28] Denu Z, Defar A, Persson L, et al. Socio-economic and geographic equity in maternal health services utilization in Ethiopia: a community-based cross-sectional study. BMC Health Serv Res. 2025;25:610. doi: 10.1186/s12913-025-12639-3 40287709 PMC12032688

[29] Chi G, Zhu J. Spatial regression models for demographic analysis. Popul Res Policy Rev. 2008;27:17–42. doi: 10.1007/s11113-007-9051-8

[30] Aragie BS, Yismaw GA, Abate BJ, et al. Geospatial variations and predictors of low birth weight in sub-Saharan Africa: a geospatial modeling using evidence from demographic health survey 2015–2024. EClinicalMedicine. 2026:91. doi: 10.1016/j.eclinm.2025.103693 PMC12774685 41509614

[31] Seboka BT, Hailegebreal S, Mamo TT, et al. Spatial trends and projections of chronic malnutrition among children under 5 years of age in Ethiopia from 2011 to 2019: a geographically weighted regression analysis. J Health Popul Nutr. 2022;41:28. doi: 10.1186/s41043-022-00309-7 35790980 PMC9254552

[32] Abate BJ, Melesse AW, Brhan H, et al. Spatial variation, pooled prevalence, and factors associated with perinatal mortality in sub-Saharan Africa, evidence from demographic and health surveys 2015–2023: a geospatial regression approach. EClinicalMedicine. 2025:81. doi: 10.1016/j.eclinm.2025.103137 PMC11929057 40124954

[33] von Elm E, Altman DG, Egger M, et al. The strengthening the reporting of observational studies in epidemiology (STROBE) statement: guidelines for reporting observational studies. Lancet. 2007;370:1453–1457. doi: 10.1016/S0140-6736(07)61602-X 18064739

[34] Hazen H, Anthamatten P. An introduction to the geography of health. New York: Routledge; 2019.

[35] Ganasegeran K, Kamarudin MK, Abdul Manaf MR. Modeling accessibility to public health facilities in resource-limited settings through GIS and geo-AI applications. In: Ram Narayan Y, Muhamad Uznir U, editors. Advances in geoinformatics technologies: facilities and utilities optimization and management for smart city applications. Singapore: Springer; 2024. p. 319–348.

[36] Wondie AG. Magnitude, factors associated with cesarean delivery and its appropriateness. In: Panagiotis T, Nikolaos N, Werner R, Von Tempelhoff GF, editors. Current topics in caesarean section. London: IntechOpen; 2021. p. 83–102.

[37] Jiang R, Dong W, Bai L, et al. Which built environment factors promote urban residents’ climate change adaptive behaviors? Multi-group application of an exploratory framework via adaptive motivations’ mediation. Sustain Cities Soc. 2026;139:107214. doi: 10.1016/j.scs.2026.107214

[38] Shi J, Li J, Zhang Z. Enhancing healthcare of remote regions: optimizing mobile healthcare units planning with intermediate depots. Socioecon Plann Sci. 2026;105:102491. doi: 10.1016/j.seps.2026.102491

[39] Nilsen C, Østbye T, Daltveit AK, et al. Trends in and socio-demographic factors associated with caesarean section at a Tanzanian referral hospital, 2000 to 2013. Int J Equity Health. 2014;13:87. doi: 10.1186/s12939-014-0087-1 25319518 PMC4206704

[40] Cao P, Pan J. Understanding factors influencing geographic variation in healthcare expenditures: a small areas analysis study. Inq J Health Care Organ Provision Finance. 2024;61:00469580231224823. doi: 10.1177/00469580231224823 PMC10823849 38281114

[41] Banke-Thomas A, Wright K, Collins L. Assessing geographical distribution and accessibility of emergency obstetric care in sub-Saharan Africa: a systematic review. J Glob Health. 2018;9:010414. doi: 10.7189/jogh.09.010414 PMC6304172 30603080

[42] Kyei-Nimakoh M, Carolan-Olah M, McCann TV. Access barriers to obstetric care at health facilities in sub-Saharan Africa—a systematic review. Syst Rev. 2017;6:110. doi: 10.1186/s13643-017-0503-x 28587676 PMC5461715

[43] Ononokpono DN, Baffour B, Richardson A. Mapping maternal healthcare access in selected West African countries. Etude Popul Afr. 2020;34:5001–5015.

[44] Ouko JJO, Gachari MK, Sichangi AW, et al. Geographic information system-based evaluation of spatial accessibility to maternal health facilities in Siaya County, Kenya. Geogr Res. 2019;57:286–298. doi: 10.1111/1745-5871.12339

[45] WHO Human Reproduction Programme. Who statement on caesarean section rates. Reprod Health Matters. 2015;23:149–150. doi: 10.1016/j.rhm.2015.07.007 26278843

[46] World Health Organization. Who recommendations on intrapartum care for a positive childbirth experience. World Health Org. 2018. 30070803

[47] Tilahun WM, Simegn MB, Abate A, et al. Caesarean section delivery and its associated factors in Ghana: a multilevel analysis. PLoS One. 2025;20:e0318223. doi: 10.1371/journal.pone.0318223 39937831 PMC11819537

[48] Boerma T, Ronsmans C, Melesse DY, et al. Global epidemiology of use of and disparities in caesarean sections. Lancet. 2018;392:1341–1348. 30322584 10.1016/S0140-6736(18)31928-7

[49] Fei Z, Wang Y. Parental gender preferences and fertility ideals in China. Acta Psychol (Amst). 2026;264:106523. doi: 10.1016/j.actpsy.2026.106523 41734732

[50] Seid A, Ahmed M. Association between health insurance enrolment and maternal health care service utilization among women in Ethiopia. BMC Public Health. 2021;21:2329. doi: 10.1186/s12889-021-12105-9 34969387 PMC8719381

[51] Wang W, Temsah G, Mallick L. The impact of health insurance on maternal health care utilization: evidence from Ghana, Indonesia and Rwanda. Health Policy Plan. 2017;32:366–375. 28365754 10.1093/heapol/czw135 PMC5400062

[52] Hoxha I, Fink G. Caesarean sections and health financing: a global analysis. BMJ Open. 2021;11:e044383. doi: 10.1136/bmjopen-2020-044383 PMC8149434 34031111

