# Supplementary material for: Geospatial inequalities and determinants of caesarean section delivery in sub-Saharan Africa: a multi-country analysis
Source: Glob Health Action. 2026 Jun 18;19(1):2686564. doi: 10.1080/16549716.2026.2686564 (PMC13288711; doi:10.1080/16549716.2026.2686564)
Supplement: Supplementary file CS_rev.docx [file ZGHA_A_2686564_SM7019.docx]

**Supplementary materials**

**Supplementary file 1:** Study area map for the study of spatial variation and determinants of cesarean section delivery in SSA, DHS 2015-2024.


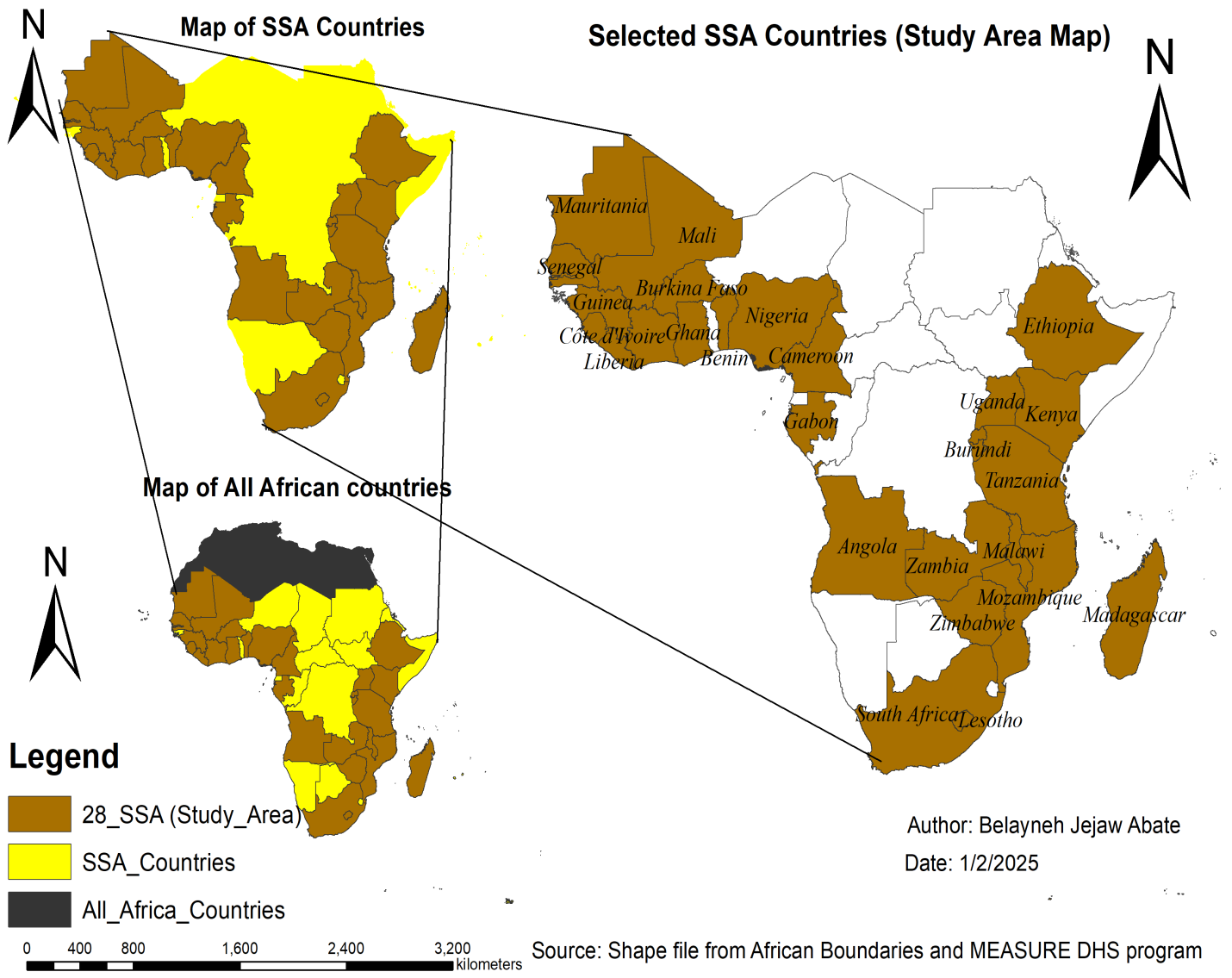


**Supplementary file 2:** Sampling procedure and sample size determination for each selected country survey, DHS 2015-2024.

Sub Saharan African Countries (SSA)

(N=46)

SSA countries that have DHS datasets

(N= 41)

SSA countries that haven’t DHS datasets

(N=5)

SSA countries that have cesarean section delivery report between 2015 and 2024

(N=28)

SSA countries that haven’t outcome report between 2015 and 2024

(N=13)

Enumeration area of selected SSA countries **(n =** 17,506**)**

Selected enumeration area that had latitude and longitude information **(n = 17,416)**

Enumeration areas don’t have latitude and longitude information (**n = 90)**

Total weighted sample of women of reproductive age who had given birth in the five years prior to the survey **(n=443,273)**

Final weighted sample size **(n=201,481)**

**Supplementary file 3:** Measurement of each predictor for the study of spatial variation and determinants of cesarean section delivery in SSA, DHS 2015-2024.

| Predictors | Category |
| --- | --- |
| Age of respondent | Less than or equal to 24 years |
|  | 25-34 years |
|  | Greater or equal to 35 years |
| Place of residence | Urban |
|  | Rural |
| Women educational attainment | No education |
|  | Primary |
|  | Secondary and above |
| Number of child in the household | 0 |
|  | 1-3 |
|  | 4 and above |
| Wealth index | Poor |
|  | Middle |
|  | Rich |
| Preceding birth interval | Less than 24 months |
|  | 24 and above |
| Birth order | 1 |
|  | 2-3 |
|  | 4 and above |
| Birth weight | Less than 2500 gram |
|  | 2500 gram and above |
| Preterm birth status | Yes |
|  | No |
| History of terminated pregnancy | Yes |
|  | No |
| Ever used anything or tried to delay or avoid getting pregnant | Yes |
|  | No |
| Age at the first birth | Less than 20 years |
|  | 20 and above years |
| Last birth by cesarean section | Yes |
|  | No |
| Antenatal care visit | No ANC |
|  | 1-3 |
|  | 4 and above |
| Source of drinking water | Improved water |
|  | Un improved water |
| Distance to health facility | No problem |
|  | Big problem |
| Media exposure | Yes |
|  | No |
| Decision making power | Yes |
|  | No |
| Insurance coverage | Yes |
|  | No |

#

# Supplementary file 4: Missing data management.

Missing data mechanisms can be classified as one of the following;

- MCAR: Missing completely at random,
- MAR: Missing at random, or
- MNAR: Missing not at random.

**1. MCAR: Missing Completely at Random**

- The probability that a value is missing is completely unrelated to any other observed or unobserved variable in the dataset. The missing data points are a random subset of the entire data.
- Example: You randomly delete 5% of the cells in your spreadsheet. The deletion doesn't depend on the row, column, or the value in the cell itself.
- The fact that the data is missing is pure chance. It's like flipping a coin to decide whether to answer a question.

**2. MAR: Missing at Random**

- The probability that a value is missing is related to other observed variables in the dataset, but not to the unobserved (missing) value itself.
- Example: In a health survey, older people are less likely to report their income. The missingness of income is related to the observed variable "age," but not to the actual, unrecorded income amount. A 70-year-old with a high income is just as likely to skip the question as a 70-year-old with a low income.
- We can predict if a value is missing based on other information we have, but not what that missing value would have been.

**3. MNAR: Missing Not at Random**

- The probability that a value is missing is related to the unobserved (missing) value itself.
- Example: In a survey about drug use, individuals who use illegal drugs are less likely to answer the question. The missingness is directly related to the very thing you are trying to measure.
- The reason the data is missing is directly linked to what the missing value would be. This is the most problematic scenario.

A preliminary assessment of data completeness was conducted across the identified predictor variables considered for analysis. This evaluation revealed that ten of these predictors contained missing observations. To quantify the extent of this issue, the proportion of missing data for each variable was systematically examined using the 'mdesc' package in Stata.

The underlying patterns of missingness were investigated in a two-stage process. First, a descriptive analysis of the frequency distributions were performed, which indicated that the missing data followed an arbitrary, non-systematic pattern across the dataset **(Table 1).** Next, a model-based assessment was performed, involving regression analyses to test whether the likelihood of missingness in a given variable was associated with observed values of other explanatory variables (majority of predictors were associated with missing value of sample predictor, which is insurance coverage as displayed in **table 2**). These analyses consistently supported classification of the missing data mechanism as Missing at Random (MAR). This means the missingness can be explained by observed data rather than unobserved values **(Table 2)**. Subsequently, missing data were managed following the procedures outlined in the Guide to DHS Statistics manual **(Table 3)**.

**Table 1**: Missing-value patterns (1 means complete)

|  | Pattern | | | | | | | | | |
| --- | --- | --- | --- | --- | --- | --- | --- | --- | --- | --- |
| Percent | 1 | 2 | 3 | 4 | 5 | 6 | 7 | 8 | 9 | 10 |
| 70% | 1 | 1 | 1 | 1 | 1 | 1 | 1 | 1 | 1 | 1 |
| 12 | 1 | 1 | 1 | 1 | 1 | 1 | 1 | 1 | 0 | 0 |
| 6 | 1 | 1 | 1 | 1 | 1 | 1 | 1 | 0 | 1 | 1 |
| 3 | 1 | 1 | 1 | 0 | 1 | 1 | 1 | 1 | 1 | 1 |
| 2 | 1 | 1 | 1 | 1 | 0 | 0 | 0 | 1 | 1 | 1 |
| 2 | 1 | 1 | 1 | 1 | 1 | 1 | 0 | 1 | 1 | 1 |
| 1 | 1 | 1 | 1 | 1 | 1 | 1 | 1 | 0 | 0 | 0 |
| <1 | 1 | 1 | 1 | 1 | 0 | 0 | 0 | 1 | 0 | 0 |
| <1 | 1 | 1 | 1 | 1 | 1 | 1 | 0 | 1 | 0 | 0 |
| <1 | 1 | 1 | 1 | 1 | 0 | 0 | 0 | 0 | 1 | 1 |
| <1 | 1 | 1 | 1 | 1 | 1 | 1 | 1 | 1 | 1 | 0 |
| <1 | 1 | 1 | 1 | 1 | 0 | 0 | 0 | 0 | 0 | 0 |
| <1 | 1 | 1 | 1 | 0 | 1 | 1 | 1 | 1 | 0 | 0 |
| <1 | 1 | 1 | 0 | 1 | 1 | 1 | 1 | 1 | 1 | 1 |
| <1 | 1 | 1 | 1 | 1 | 1 | 1 | 1 | 1 | 0 | 1 |
| <1 | 1 | 1 | 1 | 1 | 0 | 0 | 0 | 1 | 0 | 1 |
| <1 | 1 | 0 | 0 | 1 | 1 | 1 | 1 | 0 | 1 | 1 |
| <1 | 1 | 0 | 0 | 1 | 1 | 1 | 1 | 0 | 0 | 0 |
| <1 | 1 | 1 | 0 | 1 | 1 | 1 | 0 | 1 | 1 | 1 |
| <1 | 1 | 1 | 0 | 1 | 0 | 0 | 0 | 1 | 1 | 1 |
| <1 | 1 | 1 | 0 | 1 | 1 | 1 | 1 | 1 | 0 | 0 |
| <1 | 1 | 1 | 0 | 1 | 1 | 1 | 0 | 1 | 0 | 0 |
| <1 | 1 | 0 | 0 | 1 | 1 | 1 | 0 | 0 | 1 | 1 |
| <1 | 1 | 1 | 1 | 1 | 0 | 0 | 0 | 1 | 1 | 0 |
| <1 | 1 | 0 | 0 | 1 | 0 | 0 | 0 | 0 | 1 | 1 |
| <1 | 1 | 1 | 1 | 0 | 1 | 1 | 1 | 1 | 0 | 1 |
| <1 | 1 | 0 | 0 | 1 | 1 | 1 | 0 | 0 | 0 | 0 |
| <1 | 1 | 1 | 0 | 1 | 0 | 0 | 0 | 1 | 0 | 0 |
| <1 | 1 | 0 | 0 | 1 | 0 | 0 | 0 | 0 | 0 | 0 |
| <1 | 0 | 1 | 1 | 1 | 1 | 1 | 1 | 1 | 1 | 1 |
| <1 | 1 | 1 | 1 | 1 | 1 | 0 | 0 | 1 | 0 | 0 |
| 100 % |  | | | | | | | | | |

Variables are (1) frequency of reading newspaper or magazine (2) age at first birth (3) last birth by cesarean section (4) respondent's occupation (5) smokes cigarettes (6) getting permission to go (7) covered by health insurance (8) preterm birth status (9) husband/partner's occupation (10) husband/partner's educational attainment

**Table 2:** Sample regression analyses to test whether the likelihood of missingness in a given variable was associated with observed values

| miss_insurance_coverage | Coefficient | Std. err. | z | P>z | 95%conf.interval |
| --- | --- | --- | --- | --- | --- |
| Cesarean section delivery | .6731215 | .0297967 | 22.59 | 0.000 | .614721, .7315219 |
| Place of residence | -.4363568 | .0236563 | -18.45 | 0.000 | -.4827223, -.3899912 |
| Women education status | .6142629 | .0150256 | 40.88 | 0.000 | .5848132, .6437125 |
| Media exposure | .5941467 | .0240715 | 24.68 | 0.000 | .5469673, .641326 |
| Wealth index | -.4874267 | .0144336 | -33.77 | 0.000 | -.5157162, -.4591373 |
| Sex of household head | .3049943 | .0209316 | 14.57 | 0.000 | .2639691, .3460194 |
| Birth interval | -.1925243 | .0294665 | -6.53 | 0.000 | -.2502776, -.134771 |
| Birth order | -.174295 | .0171717 | -10.15 | 0.000 | -.207951, -.140639 |
| History of terminated pregnancy | -.1102202 | .0281677 | -3.91 | 0.000 | -.1654279, -.0550126 |
| Ever used anything or tried to delay or avoid getting pregnant | .2550116 | .027712 | 9.20 | 0.000 | .2006971, .3093261 |
| Wanted pregnancy | .187208 | .014731 | 12.71 | 0.000 | .1583358, .2160802 |
| ANC visit | .1194846 | .0159944 | 7.47 | 0.000 | .088136, .1508331 |
| Distance to health facility | 1.284782 | .023682 | 54.25 | 0.000 | 1.238366, 1.331198 |
| Number of children in the household | -.3556107 | .0284768 | -12.49 | 0.000 | -.4114242, -.2997972 |
| Source of drinking water | .0277531 | .021817 | 1.27 | 0.203 | -.0150075, .0705137 |
| Type of toilet facility | -.4569199 | .0219222 | -20.84 | 0.000 | -.4998866, -.4139532 |
| Using contraceptive method | .4593021 | .0251054 | 18.29 | 0.000 | .4100964, .5085078 |
| Age of respondent | .1509244 | .0203084 | 7.43 | 0.000 | .1111207, .190728 |
| Decision making power | .7760341 | .0269085 | 28.84 | 0.000 | .7232943, .8287738 |
| Birth Weight | .3133591 | .0409653 | 7.65 | 0.000 | .2330687, .3936496 |
| _cons | -4.247635 | .0887925 | -47.84 | 0.000 | -4.421665, -4.073604 |

**Table 3:** Missing data management according to guide to DHS statistics for the study of spatial variation and determinants of cesarean section delivery in SSA, DHS 2015-2024

| Variable | Missing | Total | Percent Missing | Missing data management according to guide to DHS statistics |
| --- | --- | --- | --- | --- |
| Age at first birth | 174 | 201,481 | 0.09 | A separate category for missing values, but very low percentage then replaced by median value |
| Last birth by cesarean section | 563 | 201,481 | 0.28 | Categorized in to no cesarean section delivery |
| Smokes cigarettes | 7,948 | 201,481 | 3.94 | Categorized in to no smoke cigarettes |
| Getting permission to go | 7,949 | 201,481 | 3.94 | Don't Know" and "Missing" are treated as distinct categories, but very low percentage then categorized highest mode value |
| Covered by health insurance | 5875 | 201,481 | 2.92 | A separate category for missing values, but very low percentage then categorized into high model value |
| Husband/partner's occupation | 8533 | 201,481 | 4.24 | Categorized into not working |
| Respondent's occupation | 6,237 | 201,481 | 3.09 | Categorized into not working |
| Place of residence | 0 | 201,481 | 0.00 | Don’t have missing value |
| Mother education status | 0 | 201,481 | 0.00 | Don’t have missing value |
| Media exposure | 0 | 201,481 | 0.00 | Don’t have missing value |
| Wealth index | 0 | 201,481 | 0.00 | Don’t have missing value |
| Sex of household head | 0 | 201,481 | 0.00 | Don’t have missing value |
| Birth interval | 0 | 201,481 | 0.00 | Don’t have missing value |
| Birth order | 0 | 201,481 | 0.00 | Don’t have missing value |
| History of terminated pregnancy | 0 | 201,481 | 0.00 | Don’t have missing value |
| Ever used anything or tried to delay or avoid getting pregnant | 0 | 201,481 | 0.00 | Don’t have missing value |
| Wanted pregnancy | 0 | 201,481 | 0.00 | Don’t have missing value |
| ANC visit | 0 | 201,481 | 0.00 | Don’t have missing value |
| Distance to health facility | 0 | 201,481 | 0.00 | Don’t have missing value |
| Birth size | 0 | 201,481 | 0.00 | Don’t have missing value |
| Birth weight | 0 | 201,481 | 0.00 | Don’t have missing value |
| Number of children in the household | 0 | 201,481 | 0.00 | Don’t have missing value |
| Source of drinking water source | 0 | 201,481 | 0.00 | Don’t have missing value |
| Type of toilet facility | 0 | 201,481 | 0.00 | Don’t have missing value |
| Age of respondent | 0 | 201,481 | 0.00 | Don’t have missing value |
| Decision making power of women | 0 | 201,481 | 0.00 | Don’t have missing value |
| Preterm birth status | 6,582 | 201,481 | 3.27 | Missing are replaced with most frequent category |

**Supplementary file 5:** Detailed Methodology for Spatial Analysis of Cesarean Section Delivery in Sub-Saharan Africa

1. **Global Spatial Autocorrelation (Moran's I)**

Global Moran’s I was used to assess whether the spatial distribution of C-section rates exhibited random distribution, clustering, or dispersion across survey clusters. It evaluates the extent to which similar values cluster in geographic space, indicating whether nearby survey clusters have more similar or dissimilar C-section rates than expected by chance. A statistically significant positive Moran’s I (p < 0.05) indicates spatial clustering, where high C-section rates are geographically concentrated near other high values and low rates near low values. In contrast, a significant negative value suggests spatial dispersion, reflecting a checkerboard-like pattern of dissimilar neighboring values. A non-significant result indicates a random spatial distribution with no meaningful spatial structure [1, 2].

1. **Hot Spot Analysis (Getis-Ord Gi*)**

Following confirmation of global spatial clustering, the Getis–Ord Gi* statistic was used to identify the precise geographic locations of significant high- and low-value clusters of cesarean section utilization. Unlike Global Moran’s I, which assesses overall spatial autocorrelation, Gi* evaluates each location in the context of its neighboring values to detect localized spatial concentrations. Locations with significantly high positive Z-scores were classified as hot spots, indicating clusters of elevated C-section utilizations surrounded by similarly high values. Conversely, locations with significantly low negative Z-scores were identified as cold spots, reflecting clusters of low C-section rates. Statistical significance, assessed using p-values, determined whether observed spatial clusters were unlikely to have occurred by random chance [3, 4].

1. **Cluster and Outlier Analysis (Anselin Local Moran's I)**

To further characterize spatial structure and identify local spatial heterogeneity, Local Indicators of Spatial Association (LISA), specifically Anselin’s Local Moran’s I, were applied. Unlike global measures or standard hot spot analysis, LISA detects both significant local clusters and spatial outliers by comparing each location’s value with those of its neighbors. This allows classification of each area into four distinct spatial regimes: High–High clusters, where high C-section rates are surrounded by similarly high values; Low–Low clusters, where low rates are embedded within low-rate neighbors; High–Low outliers, representing high C-section rate locations surrounded by low-rate areas; and Low–High outliers, where low-rate locations are embedded within high-rate contexts [5, 6]. This analysis is crucial for identifying not just broad clusters, but also exceptional, anomalous areas that deviate from their regional context. These outliers are critical for understanding inequities and unexpected local barriers or facilitators.

1. **Spatial Scan Statistics (SaTScan)**

SaTScan is a widely used free software designed for spatial, temporal, and space-time scan statistics to detect and evaluate statistically significant clusters in geographic data [7]. In this study, we applied the Bernoulli model because the outcome variable was binary (cesarean section delivery: yes/no). The Bernoulli model compares the spatial distribution of cases (women who had C-section delivery) and controls (women who did not have C-section delivery) across the study area. The software works by imposing scanning windows (usually circular) of varying sizes across the study area and calculating a likelihood ratio comparing observed versus expected cases inside and outside these windows under the null hypothesis of random distribution. Windows with significantly higher-than-expected proportions of C-section cases were identified as clusters. The analysis identifies the most likely and secondary statistically significant clusters and estimates their geographic location and spatial extent

1. **Ordinary Kriging interpolation**

Ordinary Kriging is a geostatistical interpolation method widely used in spatial analysis to estimate values at unsampled locations based on observed values from nearby sampled points. It assumes the spatial pattern of the variable has a constant but unknown mean across the study area and relies on modeling spatial autocorrelation through a variogram [8, 9]. In this study, Ordinary Kriging was used to generate a continuous predictive surface of C-section utilization across Sub-Saharan Africa, including areas without survey observations. This approach enabled visualization of the spatial distribution of C-section utilization across the entire region and facilitated estimation of utilization patterns in data-sparse areas, thereby supporting broader geographic assessment and informing regional health planning and resource allocation.

**Supplementary file 6:** Detailed Explanation of Spatial Regression Models

1. **Ordinary Least Squares (OLS) Regression**

Ordinary Least Squares (OLS) regression was initially applied as a global model to assess the average relationship between predictor variables (e.g., wealth status, educational level, and place of residence) and C-section rates across the study area [10]. It produces a single, global equation of the form:

CS_rate = β₀ + β₁*(Predictor₁) + β₂*(Predictor₂) + ... + ε

where each coefficient (β) represents the average expected change in the C-section rate for a one-unit change in that predictor, holding all others constant.

OLS served as the initial modeling approach for evaluating global relationships and identifying whether spatial dependence remained in the data. Residuals from the OLS model were subsequently assessed for spatial autocorrelation using Moran’s I statistic. The presence of significant spatial autocorrelation in the residuals indicates that the global model does not fully account for underlying spatial processes [7, 11], suggesting the need for more advanced spatial regression techniques, such as the Spatial Lag Model (SLM) or Spatial Error Model (SEM).

1. **Spatial Lag Model (SLM)**

The Spatial Lag Model (SLM) was used to account for spatial dependence arising from potential interactions between neighboring locations, where C-section rates in one area may be influenced by rates in surrounding areas. This model incorporates a spatially lagged dependent variable to capture potential spillover effects across geographic regions [12]. The model looks like this:

CS_rate = ρ*(W * CS_rate) + β₀ + β₁*(Predictor₁) + ... + ε

ρ (rho) is the spatial lag coefficient. It measures the strength and direction of the spillover effect.

W *C-section rate is the spatial lag, which is a weighted average of the C-section rates in neighboring locations (defined by spatial weights matrix, W). A positive and significant ρ suggests the presence of a spillover effect: high C-section rates in one region contribute to high C-section rates in its neighbors, and vice versa. This could be due to the diffusion of medical practices, patient demand, or shared cultural norms across boundaries.

1. **Spatial Error Model (SEM)**

The Spatial Error Model (SEM) was used to account for spatial dependence arising from unmeasured or omitted factors that may be spatially clustered across the study area. Unlike the Spatial Lag Model, SEM assumes that spatial dependence occurs through the error structure rather than through direct interactions between neighboring outcome values [11, 12]. The equation is:

C-section rate = β₀ + β₁*(Predictor₁) + ... + u, where u = λ*(W * u) + ε

λ (lambda) is the spatial error coefficient. It measures the degree to which the regression errors correlate across space.

u is the spatially autocorrelated error term

A significant λ indicates that the unobserved factors influencing C-section rates are clustered in space. The SEM is appropriate when the Lagrange Multiplier test for the error (LM-error) is significant, indicating that the spatial dependency is due to the model's misspecification in its error structure. The selection of an appropriate spatial regression model was guided by the decision tree presented in **Figure 1**


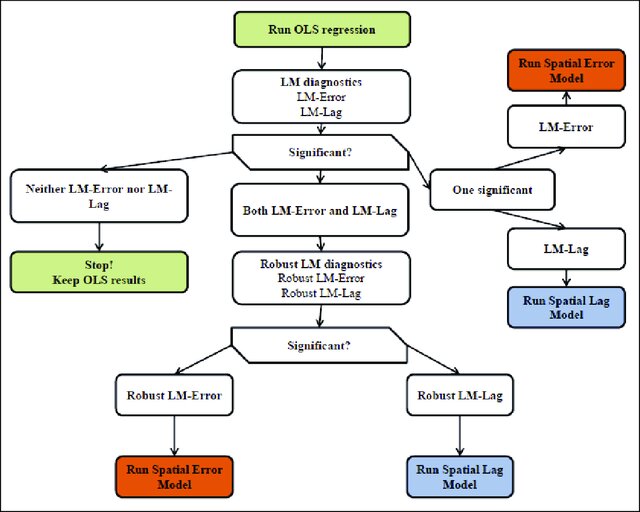


**Figure 1:** Decision tree of global regression model for determining cesarean section delivery in SSA: DHS 2015-2024 [11].

*Notes: Results are obtained from global regression model. OLS denotes ordinary least square, LM denotes Lag range multiplier.*

1. **Geographic Weighted Regression (GWR)**

Geographically Weighted Regression (GWR) was used to account for spatial non-stationarity by allowing associations between predictors and cesarean section rates to vary across geographic locations rather than assuming a single global relationship [13]. GWR estimates location-specific regression coefficients by calibrating a separate regression equation for each location, assigning greater weight to nearby observations than to distant ones according to a distance-decay function and bandwidth selection approach. This enables identification of geographic variation in the strength and direction of predictor effects.

The model can be expressed as:

yᵢ = β₀(uᵢ, vᵢ) + β₁(uᵢ, vᵢ)x₁ᵢ + β₂(uᵢ, vᵢ)x₂ᵢ + ... + εᵢ

Where (uᵢ, vᵢ) are the geographic coordinates of location i.

βₖ(uᵢ, vᵢ) is the local coefficient for predictor k at location i.

A key limitation of GWR is its assumption that all predictor variables operate at a single spatial scale through a common bandwidth. This assumption may be unrealistic because different factors may influence C-section rates at varying spatial scales, potentially leading to overfitting or reduced model accuracy.

1. **Multiscale Geographic Weighted Regression (MGWR)**

MGWR is a major advancement over GWR that relaxes the single-bandwidth assumption. It allows each relationship in the model to vary at its own unique spatial scale. This provides a more realistic and nuanced understanding of the underlying processes [14, 15]. The model uses an iterative back-fitting algorithm to optimize an independent bandwidth for each predictor variable.

The model can be expressed as:

yᵢ = β₀ (uᵢ, vᵢ) + β₁(uᵢ, vᵢ | b₁)x₁ᵢ + β₂(uᵢ, vᵢ | b₂)x₂ᵢ + ... + εᵢ

The key difference is the conditional bandwidth bₖ. Each predictor xₖ has its own optimal bandwidth bₖ that defines the spatial scale of its relationship with the outcome.

The estimated bandwidth reflects the spatial scale at which each predictor influences C-section rates. Smaller bandwidths indicate highly localized relationships with substantial spatial variation, whereas larger bandwidths suggest broader regional or near-global effects. Compared with GWR, MGWR improves model performance by reducing overfitting, minimizing distortion from imposing a common spatial scale across predictors, and providing additional insights into the geographic scale at which different determinants of CS rates operate [16].

**Supplementary file 7:** Descriptive statistics of each predictor for the study of spatial variation and determinants of cesarean section delivery in SSA, DHS 2015-2024.

| Predictors | Category | Weighted frequency (%) |
| --- | --- | --- |
| Age of respondent | Less than or equal to 24 years | 15,435 (7.66) |
|  | 25-34 years | 97,190 (48.24) |
|  | Greater or equal to 35 years | 88,856 (44.10) |
| Place of residence | Urban | 70,948 (35.21) |
|  | Rural | 130,533 (64.79) |
| Women educational attainment | No education | 69,248 (34.37) |
|  | Primary | 65,979 (32.75) |
|  | Secondary and above | 66,254 (32.88) |
| Number of child in the household | 0 | 9,539 (4.73) |
|  | 1-3 | 176,891 (87.80) |
|  | 4 and above | 15,051 (7.47) |
| Wealth index | Poor | 86,527 (42.95) |
|  | Middle | 40,103 (19.90) |
|  | Rich | 74,851 (37.15) |
| Preceding birth interval | Less than 24 months | 24,530 (12.17) |
|  | 24 and above | 176,951 (87.83) |
| Birth order | 1 | 44,877 (22.27) |
|  | 2-3 | 73,172 (36.32) |
|  | 4 and above | 83,432 (41.41) |
| Birth weight | Less than 2500 gram | 12,272 (6.09) |
|  | 2500 gram and above | 189,209 (93.91) |
| Preterm birth status | Yes | 8,006 (3.97) |
|  | No | 193,475 (96.03) |
| History of terminated pregnancy | Yes | 28,127 (13.96) |
|  | No | 173,354 (86.04) |
| Ever used anything or tried to delay or avoid getting pregnant | Yes | 112,909 (56.04) |
|  | No | 88,572 (43.96) |
| Age at the first birth | Less than 20 years | 115,711 (57.43) |
|  | 20 and above years | 85,770 (42.57) |
| Last birth by cesarean section | No | 187,414 (93.02) |
|  | Yes | 14,067 (6.98) |
| Antenatal care visit | No ANC | 22,994 (11.41) |
|  | 1-3 | 59,165 (29.36) |
|  | 4 and above | 119,322 (59.23) |
| Source of drinking water | Improved water | 136,089 (67.54) |
|  | Un improved water | 65,392 (32.46) |
| Distance to health facility | No problem | 119,260 (59.19) |
|  | Big problem | 82,221 (40.81) |
| Media exposure | Yes | 131,751 (65.39) |
|  | No | 69,727 (34.61) |
| Decision making power | Yes | 119,245 (59.18) |
|  | No | 82,236 (40.82) |
| Insurance coverage | Yes | 31,455 (15.61) |
|  | No | 170,026 (84.39) |

**Supplementary file 8:** Global spatial autocorrelation report for the study of spatial variation and determinants of cesarean section delivery in SSA, DHS 2015-2024.

**Spatial Autocorrelation Report**

| **Moran's Index** | 0.145347 |  |
| --- | --- | --- |
| **z-score** | 143.674339 | 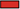 |
| **p-value** | 0.000000 |  |


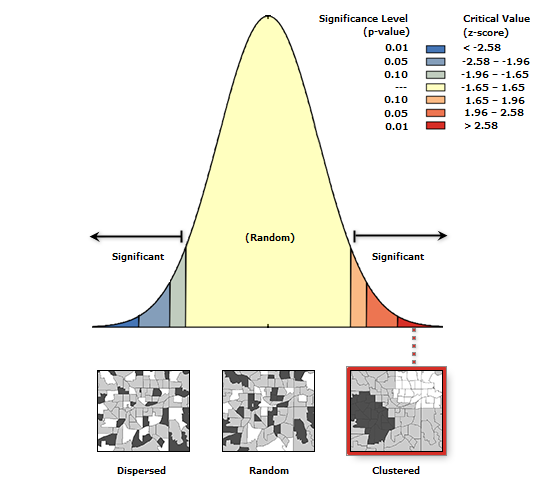


Given the z-score of 143.674339, there is a less than 1% likelihood that this clustered pattern could be the result of random chance.

**Supplementary file 9:** High low clustering report for the study of spatial variation and determinants of cesarean section delivery in SSA, DHS 2015-2024.

**High-Low Clustering Report**

| **Observed General G** | 0.000094 |  |
| --- | --- | --- |
| **z-score** | 116.780357 | 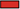 |
| **p-value** | 0.000000 |  |


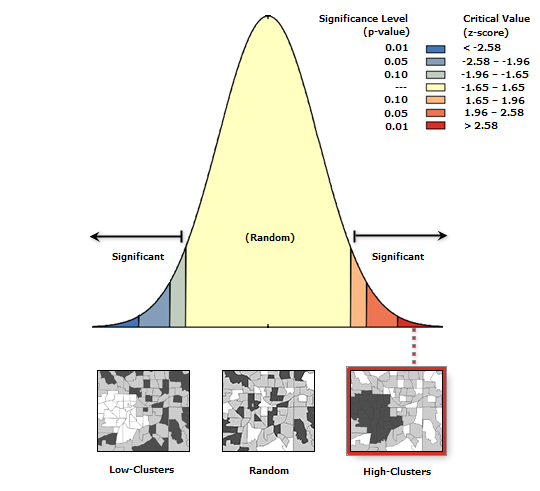


Given the z-score of 116.78035650862107, there is a less than 1% likelihood that this high-clustered pattern could be the result of random chance.

**Supplementary file 10**: Significant SatScan analysis of cesarean section delivery in SSA: DHS 2015-2024.

| Cluster type | No of location identified | Coordinates/radius km | Population | Cases | RR | LLR | P-value |
| --- | --- | --- | --- | --- | --- | --- | --- |
| Primary(most likely) | 1146 | (33.861285 S, 18.728133 E) / 1654.70 km | 4781 | 1156 | 3.7 | 715.9 | P<0.01 |
| Secondary(1^st^ cluster) | 1545 | (5.735980 S, 39.302857 E) / 762.32 km | 10282 | 1816 | 2.7 | 694.4 | P<0.01 |
| Secondary(2^nd^ cluster) | 523 | (5.691795 N, 1.280778 W) / 310.46 km | 4629 | 997 | 3.2 | 518.2 | P<0.01 |
| Secondary(3^rd^ cluster) | 456 | (16.150225 N, 16.498673 W) / 229.09 km | 5533 | 760 | 2.0 | 157.3 | P<0.01 |
| Secondary(4^th^ cluster) | 7 | (8.798992 N, 11.808725 W) / 17.96 km | 81 | 40 | 7.0 | 53.1 | P<0.01 |
| Secondary(5^th^ cluster) | 36 | (6.614866 N, 3.372534 E) / 15.20 km | 677 | 125 | 2.6 | 48.5 | P<0.01 |
| Secondary(6^th^ cluster) | 36 | (0.421678 N, 9.444671 E) / 6.93 km | 1570 | 212 | 1.9 | 40.8 | P<0.01 |
| Secondary(7^th^ cluster) | 12 | (8.480100 N, 13.268578 W) / 3.34 km | 226 | 48 | 3.0 | 23.6 | P<0.01 |
| Secondary(8^th^ cluster) | 44 | (12.303155 N, 1.574164 W) / 14.75 km | 630 | 91 | 2.1 | 20.8 | P<0.01 |
| Secondary(9^th^ cluster) | 3 | (4.764890 N, 7.025730 E) / 6.24 km | 99 | 28 | 4.0 | 20.6 | P<0.01 |
| Secondary(10^th^ cluster) | 5 | (9.144158 N, 11.645280 W) / 19.06 km | 58 | 21 | 5.2 | 20.5 | P<0.01 |
| Secondary(11^th^ cluster) | 3 | (5.525982 N, 7.017339 E) / 9.31 km | 52 | 18 | 4.9 | 16.7 | P<0.01 |
| Secondary(12^th^ cluster) | 51 | (4.494996 N, 9.566561 E) / 53.87 km | 504 | 70 | 2.0 | 14.5 | P=0.02 |

Notes: Results are obtained from SatScan analysis. RR = relative risk, LLR = log likelihood ratio

# Supplementary file 11: Model diagnosis for global regression model

Table 1: Diagnostic test for global regression models to determine factors associated with cesarean section delivery in SSA: DHS 2015-2024.

| Regression Diagnostics | | | |
| --- | --- | --- | --- |
| Multicollinarity condition number | 4.253564 | | |
| Test on normality of error | | | |
| Test | DF | Value | Probability |
| Jarque bera test | 2 | 216529.9 | 0.33263 |
| Diagnostic for heteroskedascity | | | |
| Test | DF | Value | Probability |
| Breush-Pagan test | 5 | 37448.3 | P <0.01 |
| Koenker Bassett test | 5 | 4079.4 | P <0.01 |
| Diagnostics for spatial dependence | | | |
| Test | MI/DF | Value | Probability |
| Moran’s I (error) | 0.1831 | 40.2885 | P <0.01 |
| Lagrange Multiplier (Lag) | 1 | 1579.3103 | P <0.01 |
| Robust LM (Lag) | 1 | 213.5736 | P <0.01 |
| Lagrange Multiplier (Error) | 1 | 1618.9939 | P <0.01 |
| Robust LM (Error) | 1 | 253.2572 | P <0.01 |
| Lagrange Multiplier (SARMA) | 1 | 1832.5675 | P <0.01 |

Notes: Results are obtained from regression diagnostics. MI = Moran’s I, DF = Degree of freedom

# Supplementary file 12: Performance comparison for global and local model regression

Table 1: Performance of global and local regression models for detraining cesarean section delivery in SSA, DHS 2015-2024.

| Category | OLS | SLM | SEM | GWR | MGWR |
| --- | --- | --- | --- | --- | --- |
| $\mathbf{Adjusted R}^{\mathbf{2}}$ | 50.1% | 54.4% | 54.8% | 57 | 65.1 |
| AICc | -6672.22 | -7938.41 | -7909.43 | -8932.4 | -33103.1 |

Notes: Results are obtained from global and local regression models. OLS = Ordinary Least Square, SLM = Spatial Lag Model, SEM = Spatial Error Model, GWR = Geographic Weighted Regression, MGWR = Multi-scale Geographic Weighted Regression.

**Supplementary file 13:** Summery of MGWR model result with optimal bandwidth among predictors for determining cesarean section delivery in SSA; DHS 2015-2024.

| Explanatory variable | Mean | Standard  deviation | Minimum | Median | Maximum | Optimal  Number of  Neighbors | Effective  Number of  Parameters | P value | t-statistics |
| --- | --- | --- | --- | --- | --- | --- | --- | --- | --- |
| Intercept | 0.02 | 0.3151 | -2.8631 | 0.06 | 0.779 | 68 | 713.73 | P-value <0.01 | 3.98 |
| Yes insurance coverage | 0.005 | 0.04 | -0.0867 | 0.01 | 0.084 | 2774 | 9.80 | P-value <0.01 | 2.80 |
| Age 35 and above | 0.13 | 0.144 | -0.0895 | 0.09 | 0.974 | 277 | 128.84 | P-value <0.01 | 3.55 |
| Anything used to delay pregnancy | 0.122 | 0.006 | 0.1141 | 0.126 | 0.129 | 17280 | 1.30 | 0.03 | 2.07 |
| Yes last birth by CS | 0.573 | 0.295 | -1.3175 | 0.6461 | 1.351 | 181 | 200.4 | P-value <0.01 | 3.66 |
|  | | Optimal number of neighbors | | | Effective number of parameters | | | | |
| GWR model Result | | 2.87 for each independent variables | | | 303.5 for each independent variables | | | | |

**References**

1. Chen Y. New approaches for calculating Moran’s index of spatial autocorrelation. PloS one. 2013;8(7):e68336.

2. Esri. How spatial autocorrelation (Global Moran’s I) works. URL: <https://pro> arcgis com/en/proapp/latest/tool-reference/spatial-statistics/h-how-spatialautocorrelation-moran-si-spatial-st htm. 2023.

3. Tsai P-J, Lin M-L, Chu C-M, Perng C-H. Spatial autocorrelation analysis of health care hotspots in Taiwan in 2006. BMC Public Health. 2009;9(1):464.

4. Bhunia GS, Shit PK, Maiti R. Comparison of GIS-based interpolation methods for spatial distribution of soil organic carbon (SOC). J Saudi Soc Agric Sci. 2018;17(2):114-26.

5. Seya H. Global and local indicators of spatial associations. In: Spatial analysis using big data. Elsevier; 2020. p. 33-56.

6. Tao R, Chen Y. Applying local indicators of spatial association to analyze longitudinal data: the absolute perspective. Geogr Anal. 2023;55(2):225–38.

7. Denu Z, Defar A, Persson L, Lemma S, Berhanu D, Getachew T, et al. Socio-economic and geographic equity in maternal health services utilization in Ethiopia: a community-based cross-sectional study. BMC Health Serv Res. 2025;25:610.

8. Meng Q, Liu Z, Borders BE. Assessment of regression kriging for spatial interpolation: comparisons of seven GIS interpolation methods. Cartogr Geogr Inf Sci. 2013;40(1):28–39.

9. Ikechukwu MN, Ebinne E, Idorenyin U, Raphael NI. Accuracy assessment and comparative analysis of IDW, spline and kriging in spatial interpolation of landform (topography): an experimental study. J Geogr Inf Syst. 2017;9(3):354–71.

10. Beale CM, Lennon JJ, Yearsley JM, Brewer MJ, Elston DA. Regression analysis of spatial data. Ecol Lett. 2010;13(2):246–64.

11. Chaurasia H, Srivastava S, Singh JK. Does seasonal variation affect diarrhoea prevalence among children in India? An analysis based on spatial regression models. Soc Sci Rev. 2020;118:105453.

12. Dubin RA. Spatial lags and spatial errors revisited: some Monte Carlo evidence. In: *Spatial and Spatiotemporal Econometrics*. Bingley (UK): Emerald Group Publishing Limited; 2004. doi:10.1016/S0731-9053(04)18002-X.

13. Sulekan A, Jamaludin SSS. Review on geographically weighted regression (GWR) approach in spatial analysis. Malays J Fundam Appl Sci. 2020;16(2):173–7.

14. Seboka BT, Hailegebreal S, Mamo TT, Yehualashet DE, Gilano G, Kabthymer RH, et al. Spatial trends and projections of chronic malnutrition among children under 5 years of age in Ethiopia from 2011 to 2019: a geographically weighted regression analysis. J Health Popul Nutr. 2022;41:28.

15. Abate BJ, Melesse AW, Brhan H, Agimas MC. Spatial variation, pooled prevalence, and factors associated with perinatal mortality in Sub-Saharan Africa, evidence from demographic and health surveys 2015–2023: a geospatial regression approach. EClinicalMedicine. 2025;81.

16. Fotheringham AS, Yang W, Kang W. Multiscale geographically weighted regression (MGWR). Ann Am Assoc Geogr. 2017;107(6):1247–65.
